# Lack of sleep is associated with internet use for leisure

**DOI:** 10.1371/journal.pone.0191713

**Published:** 2018-01-23

**Authors:** So Young Kim, Min-Su Kim, Bumjung Park, Jin-Hwan Kim, Hyo Geun Choi

**Affiliations:** 1 Department of Otorhinolaryngology-Head & Neck Surgery, CHA Bundang Medical Center, CHA University, Seongnam, Korea; 2 Department of Otorhinolaryngology-Head & Neck Surgery, Korea University Ansan Hospital, Ansan, Korea; 3 Department of Otorhinolaryngology-Head & Neck Surgery, Hallym University College of Medicine, Anyang, Korea; 4 Department of Otorhinolaryngology-Head & Neck Surgery, Hallym University College of Medicine, Seoul, Korea; Kent State University, UNITED STATES

## Abstract

**Objective:**

Previous studies have suggested that excessive internet use may cause lack of sleep. However, recent studies have hypothesized that lack of sleep may instigate internet use for leisure. To elucidate the potential effects of sleep time on internet use, we explored the different associations between sleep time and internet use according to its purpose.

**Methods:**

The population-based, cross-sectional study group from the Korea Youth Risk Behavior Web-based Survey (KYRBWS) collected data from 57,425 middle school students in 2014 and 2015. Sleep time over the past 7 days was classified into the following groups: < 7 h (6 h); ≥ 7 h, < 8 h (7 h); ≥ 8 h, < 9 h (8 h); and ≥ 9 h (9+ h). Internet use time per day was separately surveyed for leisure and for study and categorized as follows: 0 h; > 0 h, ≤ 1 h (1 h); > 1 h, ≤ 2 h (2 h); and > 2 h (2+ h) per day. Information on age, sex, region of residence, body mass index (BMI), economic level, parental education level, stress level, school performance level, and sleep satisfaction were retrieved. The relationships between sleep time and internet use time for leisure/study were analyzed using multinomial logistic regression with complex sampling. In the subgroup analysis according to sleep satisfaction (good, normal, and poor), the associations of sleep time with internet use for leisure were analyzed using the same methods.

**Results:**

Compared to 9+ h of sleep, less sleep was related to a long internet use time (2+ h) for leisure (adjusted odds ratio, AOR [95% confidence interval, CI] of sleep: 8 h = 1.23 [1.14–1.32]; 7 h = 1.42 [1.31–1.54]; and 6 h = 1.56 [1.44–1.70]; P < 0.001). Conversely, a relationship between less sleep and a long internet use time (2+ h) for study was evident only for 6 h of sleep (AOR of sleep: 8 h = 0.84 [0.84–1.04]; 7 h = 1.05 [0.94–1.17]; and 6 h = 1.32 [1.27–1.59]; P < 0.001). In the subgroup analysis according to sleep satisfaction, less sleep was associated with a long internet use time for leisure in all sleep satisfaction groups, although the relationship was more significant in the lower sleep satisfaction group.

**Conclusion:**

Less sleep was significantly related to long-term use of the internet for leisure, whereas this association was not definite for internet use for study. Furthermore, poor sleep quality potentiated the relationship between less sleep time and internet use for leisure.

## Introduction

The internet is an inevitable element in civilized life. More than 90% of the Korean adolescent population is estimated to surf the internet using browsers [1]. Although the internet plays an essential role in daily life, the enormous amount of content and linked pages provide an easy distraction from planned professional destinations online [2]. Diverse attractive online content and interactive communication systems facilitate falling into internet use for leisure even when the drawbacks of this use are rationally known. Internet use for leisure diminishes productivity at work, resulting in a huge economic burden [3]. Thus, elucidating its related conditions and controlling internet use for leisure are important goals.

In modernized society, many adolescents suffer from a chronic lack of sleep [4,5]. The average sleep time for American adolescents is less than 6.5 h on school days [6]. Korean adolescents sleep an estimated 5 h per day, which is considerably lower than the recommended sleep time of 7.5 to 8.5 h per day [6,7]. Prior studies have demonstrated relationships between excessive internet use and poor sleep quality [1,8]. In adolescents, frequent bedtime internet use is inversely related to sleep duration [9,10]. Generally, excessive internet use is believed to cause physical sleep deprivation. Long internet use times induce a relative shortness of sleep time, daytime fatigue, sleepiness, and arousal problems in adolescents [11,12]. Stimulating and lascivious content on the internet may be recalled at nighttime, thereby potentially causing overexcitement or nightmares [13]. Moreover, lengthening the time of light exposure can disturb circadian rhythms [11]. The reciprocal associations among risk behaviors of sleep deprivation, including internet use, alcohol and drug abuse, and use of mobile phones, were suggested to have an effect on the relationship between sleep deprivation and frequent internet use [14].

However, recent studies on these passive roles of sleep in relation to internet use are conflicting in terms of the active effect of sleep on internet use time. Psychological or other emotional conditions subsequent to sleep deprivation have been proposed to be active triggers of prolonged internet use. Indeed, sleep problems have been suggested to cause internet overuse and internet use for leisure [15–17]. These studies have proposed that sleep deprivation results in the depletion of self-regulation, thereby facilitating loafing on internet browsers not related to work [16].

Most previous studies on internet use and sleep did not consider differences regarding the purpose of internet use due to the difficulty in investigating this topic. Thus, the present study investigated the relationship between sleep time and internet use for leisure by discriminating this purpose from use for study. The running hypothesis of this study was that sleep deprivation induced internet use for leisure. In particular, the impact of sleep deprivation on internet use for leisure may be higher in the poor sleep satisfaction group than in the good sleep satisfaction group. Few studies have addressed the numerous possible confounders for sleep and internet use, such as socioeconomic factors. Socioeconomic factors, including parental factors, have been related to problematic internet use in adolescents [18,19], and body mass index (BMI) has been associated with internet use in adolescents [20]. In addition, school performance has been associated with internet use time [21]. To minimize possible bias, this study considered various confounders, including BMI, region of residence, economic level, parental education level, stress level, and school performance. We used a huge population-based representative nationwide study group, and we specifically focused on an adolescent population vulnerable to internet overuse [22]. The aim of this study was to investigate the different associations between sleep time and internet use times according to the purpose of use (for study/for leisure).

## Materials and methods

### Study population and data collection

The Institutional Review Board of the Centers for Disease Control and Prevention of Korea (KCDC) approved this study (2014-06EXP-02-P-A). Written informed consent was obtained from each participant prior to the survey. Because this web-based survey was performed at schools with a huge number of participants, informed consent from their parents was exempted. This consent procedure was approved by the Institutional Review Board of the KCDC.

This cross-sectional study used data from the Korea Youth Risk Behavior Web-based Survey (KYRBWS). This study covers one nation using statistical methods based on designed sampling and adjusted weighted values. Data from the Korea National Health and Nutrition Examination Survey (KNHANES) conducted in 2014 and 2015 were analyzed. The data were collected by the Centers for Disease Control and Prevention of Korea. Korean adolescents from the 7^th^ through 12^th^ grades completed the self-administered questionnaire voluntarily and anonymously. The validity and reliability of the KYRBWS have been documented by other studies [23,24]. The surveys evaluated data from South Korean adolescents using stratified two-stage (schools and classes) clustered sampling based on data from the Education Ministry. The sampling was weighted by statisticians, who performed post-stratification and considered the non-response rates and extreme values. In the study data, we excluded high school participants (10^th^ through 12^th^ grades). In Korea, high school students have very limited free time to access the internet due to their long school attendance times and intense academic workloads.

**Fig 1:**
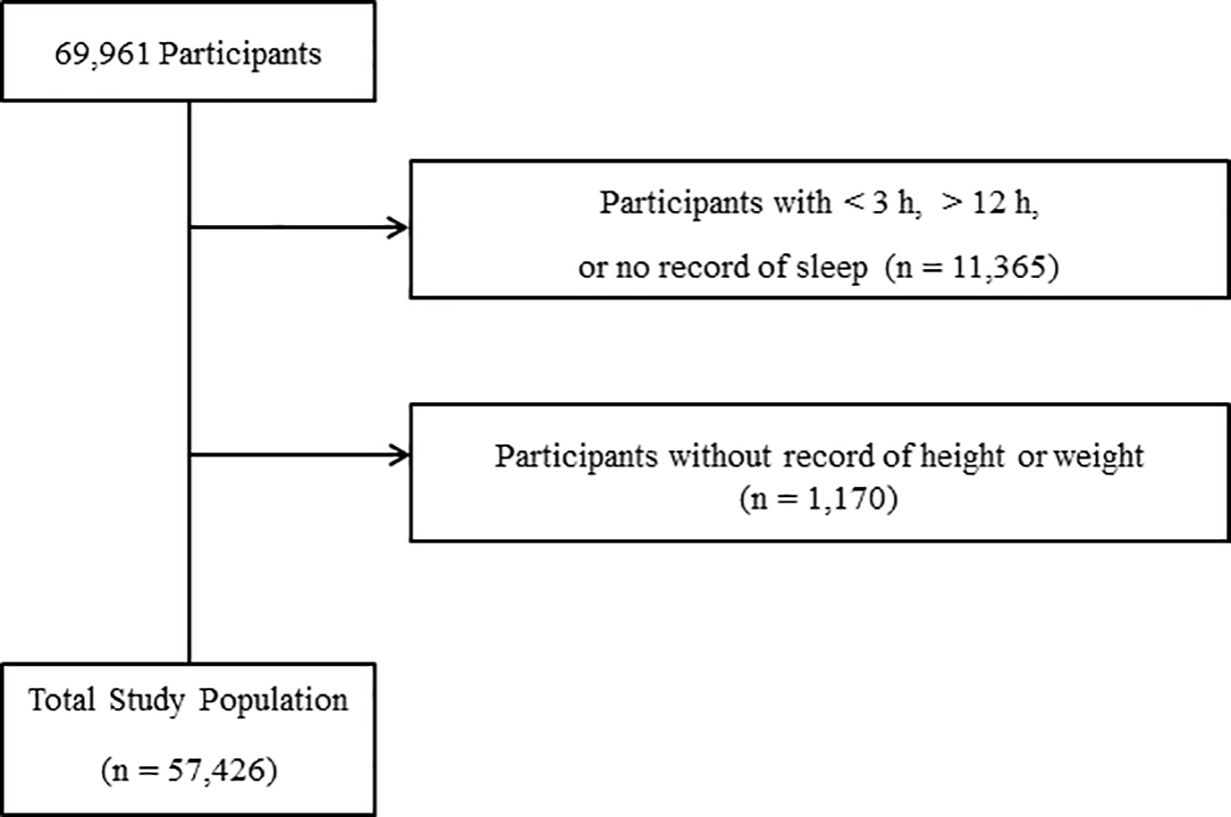
A schematic illustration of participant selection in the present study. Among a total of 69,961 participants, participants without information on sleep time or who sleep less than 2 h (11,365) and those without height or weight data (1,170) were excluded. Complete data for 57,426 participants were obtained and analyzed.

Among a total of 69,961 middle school students (7^th^ through 9^th^ grades), we excluded the following participants from this study: participants who did not complete the sleep time items or who slept less than 2 h (11,365 participants) and participants who did not record their height or weight (1,170 participants). Finally, 57,426 participants (28,863 males and 28,563 females) from 12 to 15 years old were included in this study (Fig 1).

### Survey

The understanding, reliability, and validity of each question were investigated by the KCDC to qualify the surveys [25]. The region of residence was divided into 3 groups by administrative district (large city, small city, and rural area). Obesity was categorized into 4 groups according to the Centers for Disease Control and Prevention guidelines for BMI (kg/m^2^) for children and teens [26] as follows: obese ≥ 95^th^ percentile; overweight ≥ 85^th^ percentile and < 95^th^ percentile; healthy weight ≥ 5^th^ percentile and < 85^th^ percentile; and underweight < 5^th^ percentile. The self-reported economic level was measured as 5 levels from highest to lowest. The parents' education levels were divided into the following 4 groups: college graduate or further education; graduated high school; graduated middle school or under; and unknown or no parents. Those participants who did not know the educational levels of their parents or who had no parents were not excluded because their exclusion could increase missing values for the relatively lower economic level participants. The stress levels of the participants were divided into 5 groups (severe, moderate, mild, a little, and no stress). The participants were asked about their study performances in their grades at school during the past 12 months. The performance at school was divided into the following 5 levels: A (highest); B (middle, high); C (middle); D (middle, low); and E (lowest).

Sleep time over the past 7 days was also evaluated. The falling asleep and waking up times were measured to within 10 min. The duration of sleep time was calculated by subtracting the falling asleep time from the wake-up time. The mean daily sleep time was calculated by adding the weekday and weekend sleep times with 5/7 and 2/7 weighting, respectively. Sleep time was divided into the following 4 groups: < 7 h (6 h); ≥ 7 h, < 8 h (7 h); ≥ 8 h, < 9 h (8 h); and ≥ 9 h (9+ h) [27,28]. The participants were asked whether they thought their sleep over the past 7 days was sufficient (sleep satisfaction). Sleep satisfaction was classified as very poor, poor, normal, good, and very good. Because few participants replied with extreme values (very poor or very good), we regrouped this metric into 3 groups as follows: poor (very poor and poor), normal, and good (good and very good).

The participants were asked about their internet use time for study and for leisure. The internet use times for study and for leisure were separately estimated. The mean daily internet use time was calculated by adding the weekday use time and weekend use time with 5/7 and 2/7 weighting, respectively. Internet use time was grouped into 4 units as follows: 0 h a day (0 h); > 0 h, ≤ 1 h a day (1 h); > 1 h, ≤ 2 h a day (2 h); and > 2 h a day (2+ h). Internet use for leisure was measured on the same scale and grouped into the following 4 units: 0 h a day (0 h); > 0 h, ≤ 1 h a day (1 h); > 1 h, ≤ 2 h a day (2 h); and > 2 h a day (2+ h).

### Statistical analysis

The differences in general characteristics according to the sleep time were calculated using linear regression analysis with complex sampling and the chi-square test with the Rao-Scott correction.

Adjusted odds ratios (AORs) of sleep time (independent variable) for internet use for study/leisure (dependent variable) were calculated using multinomial logistic regression analyses accounting for the complex sampling. The BMI, region of residence, economic level, parental education level, stress level, and school performance were adjusted as confounders. Multinomial logistic regression is an extension of logistic regression for when a nominal outcome has more than two unordered categories/levels. In this analysis, internet use time for leisure = 0 and internet use time for study = 0 were set as the references.

For the subgroup analysis according to sleep satisfaction, the AORs of sleep time for internet use for leisure were calculated using a multiple logistic regression analysis accounting for the complex sampling.

Two-tailed analyses were conducted, and *P*-values lower than 0.05 were considered to indicate significance. The 95% confidence intervals (CIs) were calculated. After applying the weighted values recommended by KYRBWS, all the results are presented as weighted values. The results were analyzed statistically using SPSS ver. 21.0 (IBM, Armonk, NY, USA).

## Results

Among the participants, 26.0% (14,946), 31.1% (17,862), 29% (16,641), and 13.9% (7,977) slept for 6 h, 7 h, 8 h, and 9+ h, respectively (Table 1). Each type of internet use (for study or for leisure) showed significant differences among the sleep time groups (each P < 0.001). Age, sex, region of residence, BMI, economic level, parental education level, stress level, school performance, and sleep satisfaction were significantly different according to the sleep time (each P < 0.001). Thus, these variables were adjusted as confounders to analyze the relationships between sleep time and internet use time for leisure or study.

**Table 1:** General characteristics of the participants according to sleep time.

Characteristics		Sleep Time				P Value
		< 7 h	≥ 7 h, < 8 h	≥ 8 h, < 9 h	≥ 9 h	
Total Number (n, %)		14,946 (26.0)	17,862 (31.1)	16,641 (29.0)	7977 (13.9)	
Mean Age (y)		13.7	13.6	13.4	13.3	<0.001*
Sex (n, %)						<0.001†
	Male	5,818 (20.2)	8,824 (30.6)	9,397 (32.6)	4,823 (16.7)	
	Female	9,127 (32.0)	9,038 (31.6)	7,244 (25.4)	3,152 (11.0)	
Region (n, %)						<0.001†
	Large City	7,342 (28.8)	8,042 (31.5)	7,011 (27.5)	3,102 (12.2)	
	Small City	6,643 (24.4)	8,413 (30.9)	8,172 (30.0)	4,039 (14.8)	
	Rural Area	961 (20.6)	1,407 (30.2)	1,458 (31.3)	836 (17.9)	
BMI Group						<0.001†
	Underweight	709 (23.8)	854 (28.7)	905 (30.4)	510 (17.1)	
	Healthy	1,1872 (26.4)	13,962 (31.1)	12,989 (28.9)	6,084 (13.5)	
	Overweight	1,728 (24.9)	2,217 (32.0)	2,009 (29.0)	981 (14.1)	
	Obese	637 (24.4)	829 (31.8)	738 (28.3)	402 (15.4)	
Economic Level (n, %)						<0.001†
	Highest	1,654 (27.0)	1,778 (29.0)	1,725 (28.2)	967 (15.8)	
	Middle High	4,557 (26.4)	5,279 (30.6)	5,059 (29.3)	2,365 (13.7)	
	Middle	6,692 (24.8)	8,606 (31.9)	7,994 (29.7)	3,661 (13.6)	
	Middle Low	1,665 (29.0)	1,884 (31.7)	1,600 (26.9)	803 (13.5)	
	Lowest	378 (33.3)	314 (27.6)	263 (23.2)	181 (15.9)	
Education, Father (n, %)						<0.001†
	Unknown	3,198 (21.9)	4,384 (30.0)	4,575 (31.3)	2,452 (16.8)	
	Middle School	278 (26.1)	326 (30.6)	297 (27.9)	163 (15.3)	
	High School	3,405 (24.3)	4,462 (31.9)	4,201 (30.0)	1,930 (13.8)	
	College or over	8,065 (29.1)	8,690 (31.3)	7,568 (27.3)	3,432 (12.4)	
Education, Mother (n, %)						<0.001†
	Unknown	3,063 (21.8)	4,192 (29.8)	4,417 (31.4)	2,410 (17.1)	
	Middle School	241 (26.8)	262 (29.1)	260 (28.9)	137 (15.2)	
	High School	4,324 (25.0)	5,549 (32.0)	5,165 (29.8)	2,279 (13.2)	
	College or over	7,318 (29.1)	7,859 (31.3)	6,799 (27.1)	3,151 (12.5)	
Stress Level (n, %)						<0.001†
	No	376 (15.1)	638 (25.7)	904 (36.4)	567 (22.8)	
	A Little	1,997 (17.5)	3,520 (30.9)	4,007 (35.2)	1,864 (16.7)	
	Mild	6,006 (24.1)	8,037 (32.3)	7,470 (30.0)	3,367 (13.5)	
	Moderate	4,717 (33.0)	4,479 (31.3)	3,413 (23.9)	1,687 (11.8)	
	Severe	1,850 (42.3)	1,188 (27.1)	847 (19.4)	492 (11.2)	
Performance at School (n, %)						<0.001†
	A	2,586 (29.8)	2,730 (31.5)	2,346 (27.1)	1,002 (11.6)	
	B	4,064 (26.2)	5,114 (32.9)	4,432 (28.5)	1,922 (12.4)	
	C	3,598 (23.7)	4,654 (30.6)	4,704 (31.0)	2,231 (14.7)	
	D	3,155 (24.7)	3,907 (30.6)	3,755 (29.4)	1,954 (15.3)	
	E	1,543 (29.3)	1,457 (27.6)	1,404 (26.6)	868 (16.5)	
Internet Use for Study						<0.001†
	0 h	6,956 (25.3)	8,411 (30.6)	8,147 (29.6)	3,981 (14.5)	
	> 0 h, ≤ 1 h	4,195 (24.1)	5,587 (32.2)	5,187 (29.9)	2,407 (13.9)	
	> 1 h, ≤ 2 h	2,020 (27.6)	2,332 (31.9)	2,024 (27.7)	940 (12.8)	
	> 2 h	1,775 (33.9)	1,532 (29.2)	1,283 (24.5)	649 (12.4)	
Internet Use for Leisure						<0.001†
	0 h	4,041 (26.5)	4,543 (29.8)	4,344 (28.4)	2,341 (15.3)	
	> 0 h, ≤ 1 h	3,417 (24.4)	4,353 (31.0)	4,297 (30.6)	1,960 (14.0)	
	> 1 h, ≤ 2 h	2,678 (23.5)	3,599 (31.5)	3,516 (30.8)	1,626 (14.2)	
	> 2 h	4,810 (28.8)	5,367 (32.1)	4,484 (26.8)	2,050 (12.3)	
Satisfaction with Sleep						<0.001†
	Bad	7,378 (43.1)	5,284 (30.7)	3,068 (17.9)	1,427 (8.3)	
	Normal	4,956 (25.0)	6,930 (35.0)	5,594 (28.2)	2,339 (11.8)	
	Good	2,612 (12.8)	5,684 (27.7)	7,979 (38.9)	4,211 (20.06)	

* Linear regression analysis with complex sampling, Significance at P < 0.05

† Chi-square test with Rao-Scott correction, Significance at P < 0.05

Compared to the student who reported 9+ h sleep, less sleep was related to long internet use (2+ h) for leisure (AOR [95% CI] of sleep 6 h = 1.56 [1.44–1.70]; P < 0.001). Less sleep was also associated with internet use for leisure in the 2 h and 1 h groups (P < 0.001). Conversely, a relationship between less sleep and long internet use (2+ h) for study was not evident. The relationship between sleep time and internet use for study showed significance only between internet use for 2+ h and sleep for 6 h (Table 2).

**Table 2:** Multinomial logistic regression analyses with complex sampling of sleep time for internet using time for leisure and study (Reference = internet use for leisure, 0 h; internet use for study, 0 h).

Sleep Time		Internet Use Time for Leisure, AOR (95% CI)			P-value
		> 0 h, ≤ 1 h (1 h)	> 1 h, ≤ 2 h (2 h)	> 2 h (2+ h)	
	< 7 h (6 h)	1.04 (0.95–1.13)	1.06 (0.97–1.16)	1.56 (1.44–1.70)	< 0.001*
	≥ 7 h, < 8 h (7 h)	1.09 (1.01–1.19)	1.12 (1.02–1.22)	1.42 (1.31–1.54)	
	≥ 8 h, < 9 h (8 h)	1.17 (1.08–1.27)	1.18 (1.08–1.28)	1.23 (1.14–1.32)	
	≥ 9 h (9+ h)	1	1	1	
Sleep Time		Internet Use Time for Study, AOR (95% CI)			P-value
		> 0 h, ≤ 1 h (1 h)	> 1 h, ≤ 2 h (2 h)	> 2 h (2+ h)	
	< 7 h (6 h)	0.98 (0.01–1.05)	1.06 (0.97–1.17)	1.32 (1.27–1.59)	< 0.001*
	≥ 7 h, < 8 h (7 h)	1.05 (0.98–1.13)	1.05 (0.96–1.15)	1.05 (0.94–1.17)	
	≥ 8 h, < 9 h (8 h)	1.00 (0.93–1.07)	0.94 (0.86–1.03)	0.94 (0.84–1.04)	
	≥ 9 h (9+ h)	1	1	1	

* Significance at P < 0.05

For the respondents with less satisfactory sleep, the relationships between sleep time and 2+ h internet use for leisure demonstrated higher AORs with a dose-response relationship (Table 3). The poor sleep satisfaction group showed the most marked correlation between sleep time and 2+ h internet use for leisure in a dose-dependent manner (P < 0.001). In the normal sleep satisfaction group, 2+ h internet use for leisure was less associated with the sleep time than in the poor sleep satisfaction group. The relationships were the most alleviated in the good sleep satisfaction groups.

**Table 3:** Subgroup analysis between sleep time and internet use time for leisure using multinomial logistic regression analyses with complex sampling (Reference = internet use for leisure, 0 h) according to sleep satisfaction (Poor, Normal, and Good).

**Sleep Satisfaction: Poor**					
Sleep Time		Internet Use Time for Leisure, AOR (95% CI)			P-value
		> 0 h, ≤ 1 h (1 h)	> 1 h, ≤ 2 h (2 h)	> 2 h (2+ h)	
	< 7 h (6 h)	1.09 (0.92–1.30)	1.15 (0.97–1.36)	1.72 (1.46–2.02)	< 0.001*
	≥ 7 h, < 8 h (7 h)	1.19 (1.01–1.41)	1.28 (1.08–1.53)	1.60 (1.36–1.88)	
	≥ 8 h, < 9 h (8 h)	1.18 (0.97–1.42)	1.33 (1.09–1.61)	1.39 (1.17–1.66)	
	≥ 9 h (9+ h)	1	1	1	
**Sleep Satisfaction: Normal**					
Sleep Time		Internet Use Time for Leisure, AOR (95% CI)			P-value
		> 0 h, ≤ 1 h (1 h)	> 1 h, ≤ 2 h (2 h)	> 2 h (2+ h)	
	< 7 h (6 h)	1.00 (0.86–1.16)	1.10 (0.93–1.30)	1.44 (1.24–1.67)	< 0.001*
	≥ 7 h, < 8 h (7 h)	1.12 (0.97–1.29)	1.16 (0.99–1.36)	1.42 (1.22–1.65)	
	≥ 8 h, < 9 h (8 h)	1.17 (1.03–1.34)	1.23 (1.05–1.44)	1.21 (1.04–1.40)	
	≥ 9 h (9+ h)	1	1	1	
**Sleep Satisfaction: Good**					
Sleep Time		Internet Use Time for Leisure, AOR (95% CI)			P-value
		> 0 h, ≤ 1 h (1 h)	> 1 h, ≤ 2 h (2 h)	> 2 h (2+ h)	
	< 7 h (6 h)	1.02 (0.88–1.18)	0.94 (0.81–1.10)	1.39 (1.20–1.60)	< 0.001*
	≥ 7 h, < 8 h (7 h)	0.98 (0.87–1.10)	0.97 (0.85–1.10)	1.23 (1.09–1.38)	
	≥ 8 h, < 9 h (8 h)	1.15 (1.02–1.29)	1.09 (0.97–1.22)	1.17 (1.04–1.31)	
	≥ 9 h (9+ h)	1	1	1	

* Significance at P < 0.05

## Discussion

The present study demonstrated that less sleep time was significantly correlated with more than 2 h of internet use for leisure. Conversely, less sleep time showed only a tiny association with internet use for study. Few studies have considered the effects of sleep deprivation on internet use for leisure. A recent study suggested that sleep time deprivation and poor sleep quality caused employees to use the internet for leisure [16]. These authors noted that the shift in sleep time due to daylight savings time also resulted in elevated internet use for leisure. Moreover, a short sleep time and interrupted sleep were positively associated with time spent on internet use for leisure in undergraduate students [15]. The present study is in line with this previous study and expands the relationship between sleep time and internet use for leisure by categorizing both the sleep time and internet use time. This study was pioneering, because we discriminated internet use for leisure from that for study and demonstrated a dose-response correlation between sleep deprivation and internet use time. Furthermore, sleep quality was assessed using a subgroup analysis, which confirmed a more noticeable negative relationship between sleep time in the poor sleep satisfaction group and internet use for leisure.

Several prior works have demonstrated the relationship between sleep deprivation and prolonged use of the internet or other media [29–32]. These studies presumed that excessive internet use time caused sleep deprivation by displacing sleep time [30,31]. However, the present study demonstrated that less sleep time was differentially related to internet use time in accordance with its purpose. In this study, a short sleep time was markedly more related to internet use time for leisure and less related to internet use for study. Therefore, sleep-deprived subjects spent more time on the internet for leisure but not for study. In other words, excessive internet use does not always result in sleep deprivation. Thus, the excessive overall time of internet use, irrespective of its purpose and the subsequent shortage of sleep time, perturbation of circadian rhythms, and poor sleep hygiene, cannot fully explain the relationship between sleep time and internet use time.

To explore the implications of sleep displacement by internet use time, the present study conducted a subgroup analysis according to the quality of sleep. As a result, the poor sleep satisfaction group showed stronger correlations with internet use for leisure. The good sleep satisfaction group was less correlated with internet use for leisure, although their sleep time was less than 7 h. Conversely, compared to the good sleep satisfaction group, the poor sleep satisfaction group had a higher correlation with internet use for leisure even though they slept for a sufficient time. Thus, in addition to sleep time, sleep quality is also engaged in the relationship between sleep and internet use for leisure.

The ego depletion model may provide a link between sleep deprivation and internet use for leisure [16]. Wandering internet networking not related to work is explained as a type of behavior in accordance with the depletion of self-regulation. Self-regulation is a limited resource that is spent to regulate thinking, emotions, and actions. Sleep is important for restoring self-regulation [33,34]. Thus, subjects with a shortage of sleep time fail to replenish their self-control resources [16,35]. The resultant low self-control can induce uncontrolled behavior. For example, lack of sleep has been proposed to be associated with delinquency [33].

Our results on the relationship between less sleep time and more internet use for leisure were fitted to the ego depletion model. Similar to the previous study, sufficient sleep was negatively associated with internet use for leisure by reducing stress and recharging mental wellness, such as self-esteem and autonomy [1]. Moreover, the effects of sleep deprivation on internet use for leisure could be mediated by other emotional factors. Lack of sleep may cause emotional problems, including depressive moods, confusion, anxiety, and fatigue [36]. In particular, adequate sleep time is a crucial factor in adolescents for physical development and other aspects of development [37]. Insufficient sleep in adolescents is associated with a wide range of psychological and behavioral problems, including depression [28,38]. These negative moods can induce more internet use solely for leisure. Furthermore, insomniac subjects may be more likely to be exposed to the opportunity for internet use to fill in their time [15,17].

However, an inverse influence of internet use on sleep is still possible, regardless of its purpose. Because increased internet use may eliminate time for sleep physically, the absolute internet use time for study and for leisure may have decreased the sleep time in the present study. In addition, the content of internet for leisure can influence sleep hygiene [32]. Compared to online educational content, internet games, attractive commercial advertisements, and blogs usually harbor exciting, fast-paced, and stimulating content. Indirectly, extensive exposure to these types of internet content affects mood problems, such as violence, compulsiveness, and anxiety, which mediate sleep problems [8,39,40]. Taken together, vicious cycles between sleep deprivation and internet use for leisure can be presumed in which sleep time becomes physically shortened as more time is spent on the internet browsing. Inversely, the present results imply detrimental effects of insufficient sleep on increasing internet use for leisure.

The present study is superior to previous works in several aspects. We used a huge population-based representative study group, which was collected using random nationwide sampling. Numerous factors potentially related to sleep and internet use were comprehensively investigated and adjusted using a multiple logistic regression analysis. Moreover, to the best of our knowledge, quantitative measures and classifications of sleep and internet use time have not been used in other studies. These classifications enable delineation of the effects of sleep time and internet use time in detail and are not limited to extreme cases of lack of sleep and internet addiction. Foremost, we discriminate between using the internet for study or for leisure, which provided supporting evidence that internet use for leisure but not for study was related to less sleep time, because the overall length of internet use could not explain the sleep deprivation.

However, the present study design still confines the interpretation of the causal relationship between sleep time and internet use for leisure. Due to the cross-sectional study design, we could not completely exclude possible effects of internet use on sleep time. The lack of objective parameters and dependence on a subjective survey pose a possible recall bias. We selected sleep time to represent sleep deprivation; daily time napping, medication history, and other parameters of sleep quality, including insomnia, sleepwalking, periodic leg movements, and sleep apnea, were not accounted for in the present study. The amount of internet use was measured by the time of internet use; internet-related symptoms and addictive behaviors were not assessed in this study. A future study with a longitudinal design considering various factors of sleep quality will solve the current limitations of this study and elucidate the causality between sleep problems and internet use, which has a great impact, particularly in the adolescent population.

Lack of sleep is related to internet use for leisure in Korean adolescents. The correlation between internet use and sleep time was prominent when the internet was used for leisure but was weak when the internet was used for study. The poor sleep quality group demonstrated more marked relationships between internet use for leisure and less sleep time.
